# Nurse-led lifestyle counseling in Polish primary care: the effect of current health status and perceived barriers

**DOI:** 10.3389/fpubh.2024.1301982

**Published:** 2024-02-19

**Authors:** Małgorzata Znyk, Szymon Kostrzewski, Dorota Kaleta

**Affiliations:** Department of Hygiene and Epidemiology, Medical University of Lodz, Lodz, Poland

**Keywords:** nurse, primary care, healthy lifestyle, counseling, Poland

## Abstract

**Introduction:**

Our study included counseling on diet and physical activity, smoking, e-cigarette use, and alcohol consumption. The aim was to examine the correlates of counseling provided by primary care nurses with the health status/health behaviors of nurses and the barriers in the advice provided.

**Materials and methods:**

In 2022, we conducted a cross-sectional survey among 331 nurses employed in the primary care sector in Lodz. The questionnaire consisted of three sections: characteristics of the patient population receiving treatment and provided with healthy lifestyle counseling by nurses, barriers to the process of assessing, controlling, and guiding patients, and health status/health behaviors of nurses.

**Results:**

Eighty percent of the nurses in our study provided advice on diet and physical activity to primary care patients. Over 70% of the survey participants performed minimal anti-smoking interventions forsmokers, 67.7% for alcohol drinkers, and 56.8% for e-cigarette users. The correlates of counseling in the field of diet and physical activity turned out to be the knowledge and skills, which enabled nurses to provide advice (OR = 2.57, *p* < 0.01). The correlates of the conducted minimal anti-smoking interventions in smoking patients were: subjective assessment of overweight and obesity in nurses, knowledge and skills in conducting counseling (OR = 1.92, *p* < 0.05), and measuring body weight, height and BMI (OR = 2.18, *p* < 0.01). Among the three most common barriers identified by the nurses in the process of assessing, monitoring, and guiding patients were the opinion that patients are not interested in improving their diet, physical activity, and weight loss (60.7%), lack of time (51.4%), as well as the belief that patients find it too difficult to change their current habits (54.1%).

**Conclusion:**

The results of our survey indicate that nurses’ participation in healthy lifestyle counseling in adult patients is unsatisfactory. Interventions in primary care should be designed considering the specific obstacles nurses may face in leading healthy lifestyles. Further training of nursing staff is required to increase their knowledge on healthy lifestyles.

## Introduction

The global population is aging and therefore health systems must be adapted to meet the growing demand for high-quality health care (1).

There is also an increase in the number of chronic diseases, which, along with the developing aging process, boosts the demand for health services and medical professionals (2). Among the major global public health problems are obesity and overweight, which results in the rising risk of comorbidities, mortality and generates healthcare costs (3). Similarly, smoking causes a number of tobacco-related conditions, including respiratory diseases, cancers, and cardiovascular diseases, which increase the risk of death and generate considerable healthcare costs (4).

Lifestyle routines, such as unhealthy eating habits and daily smoking, have a major impact on non-communicable diseases (5). Health authorities in several countries have issued practical recommendations and guidelines on providing lifestyle counseling in primary care (6, 7). However, healthy lifestyle counseling is carried out at a low level (8, 9). Conducting healthy lifestyle counseling is a process that aims to introduce permanent changes in the patient’s lifestyle.

The effectiveness of any healthcare system is determined by its medical staff. The efficiency of a healthcare system and the quality of medical services provided depend primarily on the knowledge, skills, and motivation of medical staff. The significant factors are the number of people authorized to practice the medical profession, the number of people actually working with patients, as well as the level of education of healthcare personnel (2).

Unfortunately, human resources in healthcare are limited, especially among physicians working in primary care. Currently, only two out of five countries in the world meet the World Health Organization’s (WHO) minimum recommendation of a physician-to-population ratio of 1:1,000 (10–12).

The nursing profession is an independent medical profession (13). Poland has been experiencing a shortage of doctors and nurses for years. In 2021, 305,800 nurses and 155,600 doctors had the right to practice in Poland (2).

The number of nurses in Poland is low compared to the neighboring countries (6.9 nurses per 1,000 Polish residents). The number of nurses in the Czech Republic is 8.9 nurses per 1,000, in Germany 14.2, in Slovakia 6.1, and in Lithuania 8.2 (14).

In the Lodz province, the number is six nurses per 1,000 residents (15). Lodz is a city located in central Poland, belonging to the Lodz Voivodeship. The number of inhabitants of the city of Lodz is 664,900, population density is 2,267 people/km^2^ (16).

In primary healthcare (PHC) units, nurses, who understand the context necessary to overcome health problems (17), are involved in prevention of complications, health promotion, and recovery, including prophylaxis and control of obesity (18).

Apart from diagnostic, nursing, rehabilitation, and treatment services, the primary care nurse also has the competence for providing health prevention (13, 19, 20).

More and more often, PHC nurses offer advice on chronic disease management and healthy lifestyle prevention (21).

Nurse counseling is a new service in primary care that combines professional competence with a new concept of health and disease management (22). The activity of primary care nurses in the field of preventive and health-promoting activities is documented in the nursing literature (23). Promoting lifestyle change requires moving from simple advice to a consultancy-based approach (24). Lifestyle risk counseling effectively supports lifestyle risk reduction and chronic disease management (25).

Shifting tasks in primary care to nurses could solve the problem of healthcare staff shortages, as well as relieve the burden on GPs (general practitioners) in providing counseling services (26). Replacing doctors with nurses is a strategy for improving the quality of care, access, and efficiency (27). It is also a way to address the shortage of healthcare workers (28).

Evidence suggests that the role of nurses could be significantly extended so that they can support primary care physicians in responding to the changing demands for health services (28).

Research results to date have shown that task shifting can address healthcare resource shortages and enable primary care physicians to provide more complex care and increase healthcare capacity (29–31). Reallocated tasks include providing care for patients with chronic diseases, health education, and prescribing medications (28).

The WHO has developed consolidated guidelines for using redeployment to address healthcare staff shortages (32). Shifting tasks to nurses allows physicians to handle more complex cases, which potentially translates into savings in staffing costs and general financial benefits for the healthcare system (33, 34).

Physical activity, healthy eating habits, and not smoking are associated with reduced cardiovascular morbidity and mortality. However, few studies have investigated the ways counseling on improving poor lifestyle habits may be provided in primary care practice.

Also, there are not many studies that have studied whether nurses’ personal factors influence their education on healthy lifestyles. It is an information gap that the authors of this paper wanted to fill. The purpose of this study was to assess the frequency of lifestyle counseling provided by primary care nurses to adult patients. Our study included counseling on diet and physical activity, smoking, e-cigarette use, and alcohol consumption. The aim was to examine the correlates of counseling provided by primary care nurses with the health status/health behaviors of nurses. Another objective of the study was to establish the frequency with which nurses measure weight, calculate body mass index (BMI), and measure waist circumference in adult patients to assess the severity of overweight or obesity. The authors also investigated what barriers nurses most often point out in the process of assessing, monitoring, and managing an adult patient, and what improvements would help reduce health problems.

## Materials and methods

### Study design and population

In 2022, we conducted a cross-sectional survey among nurses employed in the primary care sector in Lodz.

The number of nurses working in primary healthcare in the Lodz province (in units that have concluded a contract with the National Health Fund) was 1,681 (as of 31.12.2020) (35). According to data received from the National Health Fund, at the end of 2021, there were 211 primary healthcare providers in the city of Lodz. A number generator was used to draw 120 numbers (36).

From a list of 211 ranked units, 100 primary healthcare entities were selected based on the first 100 numbers drawn. In each of the 100 drawn entities, every second nurse working there was interviewed.

The researcher randomly selected every second working nurse on Monday and Tuesday mornings, and on Wednesday and Friday afternoons. If no one agreed to join the study, another third nurse was selected.

Inclusion criterion: providing services to adult patients, nurses’ voluntary and written consent to participate in the study. The survey questionnaire nurses was fully completed by 331 nurses, and incomplete questionnaire forms were rejected. Nineteen nurses refused to participate in the survey. The Bioethics Committee of the Medical University of Lodz gave a positive opinion on the research proposal (RNN/315/18/KE).

### Study variables

The nurses independently completed a paper-based survey questionnaire. It consisted of the three following sections: (1) characteristics of the patient population receiving treatment and provided with healthy lifestyle counseling by nurses; (2) barriers to the process of assessing, controlling and guiding patients; and (3) health status/health behaviors of nurses.

Socio-demographic characteristics of the nurses included age, weight, and height. Nurses were asked to subjectively asses their health status as (1) very good, (2) good, and (3) unsatisfactory.

Based on weight and height, BMI was calculated for each of the nurses. BMI (kg/m^2^) was calculated according to the following formula: weight (kg) divided by height squared (m^2^) (37). Normal-weight nurses had a BMI < 25 kg/m^2^, overweight nurses had a BMI ≥25 to <30 kg/m^2^, and obese nurses had a BMI ≥30 kg/m^2^. The subjects were also asked to subjectively assess how they were affected by overweight and obesity, as well as their intention to lose weight.

Another question referred to their existing chronic diseases (e.g., type II diabetes, hypertension, coronary artery disease, asthma, chronic obstructive pulmonary disease, and others). Responses were divided into four groups: no diseases, one, two, and three or more diseases. Nurses also provided information about existing chronic diseases in the family (diabetes mellitus, coronary artery disease, and neoplastic disease). Lifestyle questions included diet, physical activity, current smoking, e-cigarette use, and alcohol consumption. Nurses consuming an average of 400 g of vegetables and fruits in their daily diet, in at least five portions and more, were considered to adhere to the diet (38). Participants who performed 150–300 min of moderate-intensity physical activity per week or 75–150 min of high-intensity physical activity were considered physically active subjects (39). The questions included medical practice: (1) private; (2) public; years of nurses’ work: (1) < 10; (2) 10–20; and (3) > 20; and number of patient visits during a routine work week: (1) ≤ 100; (2) > 100. Additionally, nurses were asked whether they had adequate training to provide lifestyle counseling (Yes, No).

The question: “How often do you give general advice on diet, and physical activity to adult patients?” concerned counseling given by nurses. Nurses who did not give advice on healthy lifestyles marked the answer “never” to the above question. Nurses who chose the answers “always,” “sometimes” or “often” were classified as those providing counseling in the above-mentioned area.

Questions about minimal anti-smoking intervention for patients who smoke cigarettes, use e-cigarettes or use alcohol provided information about the nurse’s provision of minimal interventions in the field of a healthy lifestyle for patients. Minimal intervention is a short action taken during a doctor’s visit, which involves identifying a patient, e.g., a smoker, and providing him with professional help in quitting the addiction (40).

The questionnaire also included questions about measuring weight on a scale, waist circumference, height, patient-reported weight, and calculating body mass index in adult patients by nurses. Possible answers to be marked were “Yes” and “No.”

The questionnaire also included statements: “A nurse is obliged to provide counseling on healthy lifestyle to patients,” “Patients are more likely to introduce lifestyle changes after receiving expert counseling from a nurse,” “There are effective strategies and tools designed to help patients with healthy lifestyle,” “I have sufficient knowledge and skills to advise patients on healthy lifestyle,” “I am effective in helping my patients to lead a healthy lifestyle,” and “Healthy lifestyle counseling will be more effective if a nurse herself/himself follows the health recommendations.” Five responses were possible for each statement: “strongly disagree,” “rather disagree,” “do not know,” “strongly agree,” and “rather agree.” The first three options were considered negative (“No”), and the remaining ones positive (“Yes”).

The questionnaire took into account barriers in the process of patient evaluation, follow-up and management, and improvements that would help reduce health problems related to diet, physical activity, and body weight (questionnaire in Supplementary material). Nurses indicated the three most important barriers and the three most important improvements.

The questionnaire was created by specialists and was validated in a previous large study (41). A survey was also conducted among GPs and patients, the results were presented in the other articles (42–44).

### Statistical analysis

Descriptive statistics and the distribution of the variables studied are presented. Numbers and percentages were used to show the data. The distribution of categorical variables was presented using frequencies and proportions along with 95% confidence intervals (95% CI). The chi-square test for independent samples was used to compare categorical variables. A *p* < 0.05 was considered statistically significant. A univariate and multivariate logistic regression analysis was performed to identify correlates of preventive actions performed by the nurse. The unadjusted model used univariate regression. In univariate analysis, all variables were considered independently. For the adjusted model, only statistically significant variables in univariate analysis were included. The statistical significance level was based on the criterion *p* < 0.05. The results were presented by odds ratio (OR) and 95%CI. Variables with *p*-values of 0.05 or less from the univariate analysis were included in the multivariate model. STATISTICA 13.3 (StatSoft, licensed from the Medical University of Lodz) was used for statistical analysis.

## Results

### Characteristics of the surveyed population

Nurses among whom the largest group were women aged 55 and over (44.1%) were surveyed. The obtained data showed that 78.9% of the study participants subjectively assessed their health status as good.

Among 331 surveyed primary health care nurses from Lodz, 38.1% were overweight, and 16.9% were obese. Subjectively assessed abnormal BMI was indicated by 29% of the nurses, while 54.1% of the subjects wanted to lose weight. Also, 33.8% of the nurses declared one chronic disease, 14.2% two chronic diseases, and 21.5% three or more chronic diseases.

In the survey, 45.6% of the nurses reported doing 150–300 min of moderate-intensity or 75–150 min of high-intensity physical activity per week, whereas 42.6% of the survey participants consumed an average of 400 g of fruits and vegetables in their daily diet in at least five servings or more. Among the study group, 18.1% currently smoked cigarettes, 6% used e-cigarettes, and 41.4% consumed alcohol.

Sixty-four percent of the nurses were employed with the public medical sector being their primary place of work, 35.7% had professional experience <10 years, while 86.7% admitted up to 100 patients during a regular working week.

Among the study participants, 72.5% declared to have sufficient knowledge and skills to advise patients on a healthy lifestyle, whereas 68% took measurements of body weight, height, and BMI in adult patients. The characteristics of the study population are included in Table 1.

**Table 1:** The characteristics of the studied population of nurses.

Characteristics	Total	%
	*n = 331*	
Age (years)		
<40	47	14.2
40–54	138	41.7
55+	146	44.1
Subjective health assessment		
Very good	61	18.4
Good	261	78.9
Unsatisfactory	9	2.7
Body mass index (BMI)		
<25 kg/m^2^	149	45.0
≥25 to <30 kg/m^2^	126	38.1
≥30 kg/m^2^	56	16.9
Number of chronic diseases		
0	101	30.5
1	112	33.8
2	47	14.2
≥3	71	21.5
Diseases occurring in the family		
Diabetes mellitus	128	38.7
Coronary artery disease	141	42.6
Neoplastic disease	150	45.3
Subjective assessment of being overweight or obese		
Yes	96	29.0
No	235	71.0
Weight loss intention		
Yes	179	54.1
No	152	45.9
Physical activity		
Yes	151	45.6
No	180	54.4
Diet		
Yes	141	42.6
No	190	57.4
Medical practice		
Private	119	36.0
Public	212	64.0
Years of work		
<10	118	35.7
10–20	105	31.7
>20	108	32.6
Number of patient visits during the routine working week		
≤100	287	86.7
>100	44	13.3
Sufficient knowledge and skills to advise patients on healthy lifestyle		
Yes	240	72.5
No	91	27.5
Making measurements of body weight, height, BMI		
Yes	225	68.0
No	106	32.0
Tobacco smoking		
Yes	60	18.1
No	271	81.9
E-cigarette use		
Yes	20	6.0
No	311	94.0
Alcohol consumption		
Yes	137	41.4
No	194	58.6

### Providing counseling on healthy lifestyles by nurses

In the study, 88.5% of nurses provided counseling on changing eating habits or physical activity in patients (total) and in patients with chronic diseases, whereas 86.7% of nurses provided such counseling to “healthy” patients (Table 2).

**Table 2:** Correlates of advice given by a nurse.

Variables	Advice on changing eating habits or physical activity in patients (Total) *N = 331*						Advice on changing eating habits or physical activity in patients with chronic diseases *N = 331*					Advice on changing eating habits or physical activity in ″healthy″ patients *N = 331*				
	Unadjusted model				Adjusted model		Unadjusted model			Adjusted model		Unadjusted model			Adjusted model	
	*N*	*n* = 293 (88.5%)	OR	95% Cl	OR	95% Cl	*n* = 293 (88.5%)	OR	95% Cl	OR	95% Cl	*n* = 287 (86.7%)	OR	95% Cl	OR	95% Cl
Age (years)
<40	47	40 (85.1)	1.00	Ref.			38 (80.9)	1.00	Ref.			40 (85.1)	1.00	Ref.		
40–54	138	122 (88.4)	1.33	0.51–3.47			124 (89.9)	2.10	0.84–5.23			117 (84.8)	0.98	0.39–2.47		
55+	146	131 (89.7)	1.53	0.58–4.01			131 (89.7)	2.07	0.84–5.10			130 (89.0)	1.42	0.55–3.70		
Subjective health assessment
Very good	61	55 (90.2)	2.62	0.44–15.58			54 (88.5)	0.96	0.10–8.90			52 (85.2)	0.72	0.08–6.49		
Good	261	231 (88.5)	2.20	0.44–11.08			231 (88.5)	0.96	0.12–7.97			227 (87.0)	0.83	0.1–6.88		
Unsatisfactory	9	7 (77.8)	1.00	Ref.			8 (88.9)	1.00	Ref.			8 (88.9)	1.00	Ref.		
Body mass index (BMI)
<25 kg/m^2^	149	137 (91.9)	2.19	0.87–5.52			136 (91.3)	1.26	0.45–3.48			131 (87.9)	1.21	0.50–2.97		
≥25 to <30 kg/m^2^	126	109 (86.5)	1.23	0.51–2.95			107 (84.9)	0.68	0.25–1.80			108 (85.7)	1.00	0.41–2.46		
≥30 kg/m^2^	56	47 (83.9)	1.00	Ref.			50 (89.3)	1.00	Ref.			48 (85.7)	1.00	Ref.		
Number of chronic diseases
0	101	91 (90.1)	1.00	Ref.			89 (88.1)	1.00	Ref.			85 (84.2)	1.00	Ref.	1.00	Ref.
1	112	100 (89.3)	0.92	0.38–2.22			98 (87.5)	0.94	0.41–2.15			96 (85.7)	1.13	0.53–2.40	0.96	0.42–2.19
2	47	42 (89.4)	0.92	0.29–2.87			43 (91.5)	1.45	0.44–4.76			40 (85.1)	1.08	0.41–2.82	0.90	0.32–2.54
≥3	71	60 (84.5)	0.60	0.24–1.50			63 (88.7)	1.06	0.41–2.75			66 (93.0)	2.49	0.87–7.13^*^	1.79	0.59–5.44
Diseases occurring in the family
Diabetes mellitus																
Yes	128	114 (89.1)	1.09	0.54–2.20			120 (93.8)	2.60	1.15–5.87^**^	2.26	0.96–5.32	114 (89.0)	1.41	0.72–2.78		
No	203	179 (88.2)	1.00	Ref.			173 (85.2)	1.00	Ref.	1.00	Ref.	173 (85.2)	1.00	Ref.		
Coronary artery disease
Yes	141	124 (87.9)	0.91	0.46–1.79			131 (92.9)	2.26	1.06–4.83^*^	1.71	0.77–3.83	121 (85.8)	0.87	0.46–1.66		
No	190	169 (88.9)	1.00	Ref.			162 (85.3)	1.00	Ref.	1.00	Ref.	166 (87.4)	1.00	Ref.		
Neoplastic disease																
Yes	150	134 (89.3)	14.59	8.01–26.6^***^	1.24	0.61–2.52	130 (86.7)	0.72	0.36–1.41			131 (87.3)	1.11	0.58–2.10		
No	181	66 (36.5)	1.00	Ref.	1.00	Ref.	163 (90.1)	1.00	Ref.			156 (86.2)	1.00	Ref.		
Subjective assessment of being overweight or obese
Yes	96	85 (88.5)	1.00	0.48–2.11			84 (87.5)	0.87	0.42–1.81			84 (87.5)	1.10	0.54–2.25		
No	235	208 (88.5)	1.00	Ref.			209 (88.9)	1.00	Ref.			203 (86.4)	1.00	Ref.		
Weight loss intention
Yes	179	153 (85.5)	0.50	0.25–1.04^*^	0.49	0.23–1.03	154 (86.0)	0.58	0.28–1.17			155 (86.6)	0.98	0.52–1.85		
No	152	140 (92.1)	1.00	Ref.	1.00	Ref.	139 (91.4)	1.00	Ref.			132 (86.8)	1.00	Ref.		
Physical activity
Yes	151	132 (87.4)	0.82	0.42–1.61			133 (88.1)	0.92	0.47–1.82			127 (84.1)	0.66	0.35–1.25		
No	180	161 (89.4)	1.00	Ref.			160 (88.9)	1.00	Ref.			160 (88.9)	1.00	Ref.		
Diet																
Yes	141	125 (88.7)	1.02	0.52–2.03			126 (89.4)	1.16	0.58–2.31			124 (87.9)	1.21	0.63–2.32		
No	190	168 (88.4)	1.00	Ref.			167 (87.9)	1.00	Ref.			163 (85.8)	1.00	Ref.		
Medical practice
Private	119	104 (87.4)	1.00	Ref.			102 (85.7)	1.00	Ref.			101 (84.9)	1.00	Ref.		
Public	212	189 (89.2)	1.19	0.59–2.37			191 (90.1)	1.52	0.77–3.00			186 (87.7)	1.28	0.67–2.44		
Years of work
<10	118	106 (89.8)	1.00	Ref.			102 (86.4)	1.00	Ref.			98 (83.1)	1.00	Ref.	1.00	Ref.
10–20	105	89 (84.8)	0.63	0.28–1.40			92 (87.6)	1.11	0.51–2.43			89 (84.8)	1.14	0.55–2.33	0.84	0.38–1.84
>20	108	98 (90.7)	1.11	0.46–2.68			99 (91.7)	1.73	0.73–4.09			100 (92.6)	2.55	1.07–6.06^*^	1.96	0.79–4.90
Number of patient visits during the routine working week
≤100	287	255 (88.9)	1.26	0.49–3.21			254 (88.5)	0.99	0.36–2.68			256 (89.2)	3.46	1.64–7.31^**^	3.97	1.72–9.21
>100	44	38 (86.4)	1.00	Ref.			39 (88.6)	1.00	Ref.			31 (70.5)	1.00	Ref.	1.00	Ref.
Sufficient knowledge and skills to advise patients on healthy lifestyle
Yes	240	220 (91.7)	2.71	1.36–5.41^**^	2.57	1.28–5.19^**^	219 (91.3)	2.40	1.20–4.78^**^	2.17	1.06–4.45^*^	217 (90.4)	2.83	1.48–5.42^**^	2.41	1.19–4.91^*^
No	91	73 (80.2)	1.00	Ref.	1.00	Ref.	74 (81.3)	1.00	Ref.	1.00	Ref.	70 (76.9)	1.00	Ref.	1.00	Ref.
Making measurements of body weight, height, BMI
Yes	225	204 (90.7)	1.86	0.93–3.69^*^	1.87	0.92–3.81	207 (92.0)	2.67	1.35–5.30^**^	2.54	1.25–5.14^**^	203 (90.2)	2.42	1.27–4.60^**^	2.64	1.31–5.33^**^
No	106	89 (84.0)	1.00	Ref.	1.00	Ref.	86 (81.1)	1.00	Ref.	1.00	Ref.	84 (79.2)	1.00	Ref.	1.00	Ref.
Tobacco smoking
Yes	60	52 (86.7)	0.81	0.35–1.87			55 (91.7)	1.53	0.57–4.09			53 (88.3)	1.20	0.51–2.83		
No	271	241 (88.9)	1.00	Ref.			238 (87.8)	1.00	Ref.			234 (86.3)	1.00	Ref.		
E-cigarette use
Yes	20	19 (95.0)	2.57	0.33–19.73			19 (95.0)	2.57	0.33–19.73			19 (95.0)	3.05	0.40–23.36		
No	311	274 (88.1)	1.00	Ref.			274 (88.1)	1.00	Ref.			268 (86.2)	1.00	Ref.		
Alcohol consumption
Yes	137	121 (88.3)	0.97	0.49–1.92			123 (89.8)	1.24	0.62–2.50			120 (87.6)	1.14	0.60–2.19		
No	194	172 (88.7)	1.00	Ref.			170 (87.6)	1.00	Ref.			167 (86.1)	1.00	Ref.		

Variables	Minimal anti-smoking intervention in smoking patients (Total) *N = 331*						Minimal anti-smoking intervention in e-cigarette users *N = 331*					Minimal anti-alcohol intervention in patients who consume alcohol *N = 331*				
	Unadjusted model				Adjusted model		Unadjusted model			Adjusted model		Unadjusted model			Adjusted model	
	*N*	*n* = 235 (71%)	OR	95% Cl	OR	95% Cl	*n* = 188 (56.8%)	OR	95% Cl	OR	95% Cl	*n* = 224 (67.7%)	OR	95% Cl	OR	95% Cl
Age (years)
<40	47	37 (78.7)	1.00	Ref.			26 (55.3)	1.00	Ref.			34 (72.3)	1.00	Ref.		
40–54	138	92 (66.7)	0.54	2.45–1.18			72 (52.2)	0.88	0.45–1.71			89 (64.5)	0.69	0.34–1.44		
55+	146	106 (72.6)	0.72	0.33–1.57			90 (61.6)	1.30	0.67–2.52			101 (69.2)	0.86	0.41–1.78		
Subjective health assessment
Very good	61	43 (70.5)	0.30	0.03–2.56			32 (52.5)	0.55	0.13–2.41			38 (62.3)	0.21	0.02–1.76		
Good	261	184 (70.5)	0.30	0.04–2.43			150 (57.5)	0.68	0.17–2.76			178 (68.2)	0.27	0.03–2.18		
Unsatisfactory	9	8 (88.9)	1.00	Ref.			6 (66.7)	1.00	Ref.			8 (88.9)	1.00	Ref.		
Body mass index (BMI)
<25 kg/m^2^	149	95 (63.8)	0.64	0.33–1.27			74 (49.7)	0.51	0.27–0.96			90 (60.4)	0.72	0.38–1.38		
≥25 to <30 kg/m^2^	126	99 (78.6)	1.34	0.65–2.78			77 (61.1)	0.81	0.42–1.56			96 (76.7)	1.52	0.76–3.04		
≥30 kg/m^2^	56	41 (73.2)	1.00	Ref.			37 (66.1)	1.00	Ref.			38 (67.9)	1.00	Ref.		
Number of chronic diseases
0	101	72 (71.3)	1.00	Ref.			60 (59.4)	1.00	Ref.			69 (68.3)	1.00	Ref.		
1	112	75 (67.0)	0.82	0.46–1.46			56 (50.0)	0.68	0.40–1.18			69 (61.6)	0.74	0.42–1.31		
2	47	35 (74.5)	1.18	0.54–2.58			25 (53.2)	0.78	0.39–1.56			32 (68.1)	0.99	0.47–2.08		
≥3	71	53 (74.6)	1.19	0.60–2.36			47 (66.2)	1.34	0.71–2.52			54 (76.1)	1.47	0.74–2.93		
Diseases occurring in the family
Diabetes mellitus																
Yes	128	96 (75.0)	1.38	0.84–2.27			79 (61.7)	1.39	0.89–2.18			92 (71.9)	1.38	0.85–2.22		
No	203	139 (68.5)	1.00	Ref.			109 (53.7)	1.00	Ref.			132 (65.0)	1.00	Ref.		
Coronary artery disease
Yes	141	110 (78.0)	1.85	1.12–3.04^**^	1.66	0.98–2.79	91 (64.5)	1.75	1.12–2.73^**^	1.65	1.02–2.66^*^	105 (74.5)	1.74	1.08–2.81^*^	1.65	1.01–2.70^*^
No	190	125 (65.8)	1.00	Ref.	1.00	Ref.	97 (51.1)	1.00	Ref.	1.00	Ref.	119 (62.6)	1.00	Ref.	1.00	Ref.
Neoplastic disease
Yes	150	109 (72.7)	1.16	0.72–1.87			89 (59.3)	1.21	0.78–1.87			108 (72.0)	1.44	0.90–2.30		
No	181	126 (69.6)	1.00	Ref.			99 (54.7)	1.00	Ref.			116 (64.1)	1.00	Ref.		
Subjective assessment of being overweight or obese
Yes	96	76 (79.2)	1.82	1.03–3.19^*^	1.92	1.07–3.45^*^	62 (64.6)	1.58	0.97–2.58^**^	1.55	0.91–2.63	70 (72.9)	1.42	0.84–2.39		
No	235	159 (67.7)	1.00	Ref.	1.00	Ref.	126 (53.6)	1.00	Ref.	1.00	Ref.	154 (65.5)	1.00	Ref.		
Weight loss intention
Yes	179	132 (73.7)	1.34	0.83–2.15			104 (58.1)	1.12	0.73–1.74			127 (70.9)	1.39	0.87–2.20		
No	152	103 (67.8)	1.00	Ref.			84 (55.3)	1.00	Ref.			97 (63.8)	1.00	Ref.		
Physical activity
Yes	151	115 (76.2)	1.60	0.98–2.60^*^	1.33	0.80–2.23	93 (61.6)	1.44	0.92–2.23			108 (71.5)	1.39	0.87–2.21		
No	180	120 (66.7)	1.00	Ref.	1.00	Ref.	95 (52.8)	1.00	Ref.			116 (64.4)	1.00	Ref.		
Diet
Yes	141	96 (68.1)	0.78	0.49–1.26			80 (56.7)	0.99	0.64–1.55			92 (65.2)	0.83	0.52–1.31		
No	190	139 (73.2)	1.00	Ref.			108 (56.8)	1.00	Ref.			132 (69.5)	1.00	Ref.		
Medical practice
Private	119	83 (69.7)	1.00	Ref.			70 (58.8)	1.00	Ref.			79 (66.4)	1.00	Ref.		
Public	212	152 (71.7)	1.10	0.67–1.80			118 (55.7)	0.88	0.56–1.38			145 (68.4)	1.10	0.68–1.77		
Years of work
<10	118	83 (70.3)	1.00	Ref.			63 (53.4)	1.00	Ref.	1.00	Ref.	77 (65.3)	1.00	Ref.		
10–20	105	71 (67.6)	0.88	0.50–1.56			54 (51.4)	0.92	0.55–1.57	0.82	0.46–1.46	68 (64.8)	0.98	0.56–1.70		
>20	108	81 (75.0)	1.27	0.70–2.28			71 (65.7)	1.68	0.98–2.87^*^	1.45	0.81–2.60	79 (73.1)	1.45	0.82–2.56		
Number of patient visits during the routine working week
≤100	287	203 (70.7)	0.91	0.45–1.84			161 (56.1)	0.80	0.42–1.54			194 (67.6)	0.97	0.49–1.92		
>100	44	32 (72.7)	1.00	Ref.			27 (61.4)	1.00	Ref.			30 (68.2)	1.00	Ref.		
Sufficient knowledge and skills to advise patients on healthy lifestyle
Yes	240	181 (75.4)	2.10	1.26–3.50^**^	1.92	1.13–3.28^*^	146 (60.8)	1.81	1.11–2.95^**^	1.79	1.05–3.04^*^	175 (72.9)	2.31	1.40–3.81^***^	2.17	1.30–3.62^**^
No	91	54 (59.3)	1.00	Ref.	1.00	Ref.	42 (46.2)	1.00	Ref.	1.00	Ref.	49 (53.8)	1.00	Ref.	1.00	Ref.
Making measurements of body weight, height, and BMI
Yes	225	172 (75.4)	2.22	1.35–3.63^**^	2.18	1.30–3.66^**^	144 (64.0)	2.51	1.56–4.02^***^	2.54	1.54–4.18^***^	162 (72.0)	1.83	1.13–2.96^**^	1.75	1.06–2.87^*^
No	106	63 (59.4)	1.00	Ref.	1.00	Ref.	44 (41.5)	1.00	Ref.	1.00	Ref.	62 (58.5)	1.00	Ref.	1.00	Ref.
Tobacco smoking
Yes	60	45 (75.0)	1.28	0.67–2.42			43 (71.7)	2.20	1.19–4.05^**^	2.44	1.26–4.71^**^	46 (76.7)	1.72	0.90–3.28		
No	271	190 (70.1)	1.00	Ref.			145 (53.5)	1.00	Ref.	1.00	Ref.	178 (65.7)	1.00	Ref.		
E-cigarette use
Yes	20	13 (65.0)	0.74	0.29–1.93			12 (60.0)	1.15	0.46–2.89			12 (60.0)	0.70	0.28–1.77		
No	311	222 (71.4)	1.00	Ref.			176 (56.6)	1.00	Ref.			212 (68.2)	1.00	Ref.		
Alcohol consumption
Yes	137	96 (70.1)	0.93	0.57–1.50			80 (58.4)	1.12	0.72–1.74			94 (68.6)	1.08	0.67–1.72		
No	194	139 (71.6)	1.00	Ref.			108 (55.7)	1.00	Ref.			130 (67.0)	1.00	Ref.		

^*^p < 0.05, ^**^p < 0.01, ^***^p < 0.001; Fully-adjusted model, including all statistically significant characteristics. Ref, reference; CI, Confidence interval.

Approximately 71% of nurses conducted minimal anti-smoking interventions in patients who smoke cigarettes, 56.8% minimal anti-smoking interventions in individuals using e-cigarettes, and 67.7% conducted minimal anti-alcohol interventions in patients who consume alcohol.

### Correlates related to healthy lifestyle counseling

The association of the nurses’ personal characteristics with healthy lifestyle counseling was based on logistic regression analyses. The odds ratio (OR), 95% confidence interval (Cl) were used to measure the strength of the association. Table 2 shows the results of univariate and multivariate logistic regression analyses for counseling provided by nurses with socio-demographic and health correlates. Variables that were significantly statistically significant in univariate analysis were taken for multivariate analysis.

In a multivariate model, a higher chance of providing advice on nutrition and physical activity to adult patients (in total) was observed among nurses who had the knowledge and skills to provide lifestyle advice (OR = 2.57; 95%Cl: 1.28–5.19, *p* < 0.01).

Nurses with knowledge and skills in counseling were more than twice as likely to provide advice on nutrition and physical activity (OR = 2.17; 95%Cl: 1.06–4.45, *p* < 0.05) to patients with chronic diseases. Whereas, respondents who measured weight, height, and BMI were two and a half times more likely to provide the aforementioned counseling (OR = 2.54; 95%Cl: 1.25–5.14, *p* < 0.01).

Similarly, higher odds of counseling on diet and physical activity in “healthy” patients were observed among nurses seeing ≤100 patients per work week (OR = 3.97; 95%Cl: 1.72–9.21, *p* < 0.01), having the knowledge and skills to offer counseling in this area (OR = 2.41; 1.19–4.91, *p* < 0.05), and taking measurements of weight, height and BMI (OR = 2.64; 95%Cl: 1.31–5.33, *p* < 0.01).

Minimal anti-smoking intervention in cigarette-smoking patients was more likely to be given when a nurse’s subjective assessment indicated that they were overweight and obese (OR = 1.92; 95%Cl: 1.07–3.45, *p* < 0.05); had the knowledge and skills to provide counseling to patients (OR = 1.92; 95%Cl: 1.13–3.28, *p* < 0.05); taking measurements of weight, height and BMI (OR = 2.18; 95%Cl: 1.30–3.66, *p* < 0.01).

Similarly, nurses who were knowledgeable and skilled in e-cigarette use (OR = 1.79; 95%Cl: 1.05–3.04, *p* < 0.05), who measured weight, height, and BMI (OR = 2.54; 95%Cl: 1.54–4.18, *p* < 0.001), and who smoked tobacco (OR = 2.44; 95%Cl: 1.26–4.71, *p* < 0.01) and who had a family history of coronary artery disease (OR = 1.65; 95%Cl: 1.02–2.66, *p* < 0.05) were more likely to provide minimal anti-smoking intervention to e-cigarette users.

Nurses with a family history of coronary artery disease (OR = 1.65; 95%Cl: 1.01–2.70, *p* < 0.05), knowledge and skills in providing healthy lifestyle counseling (OR = 2.17; 95%Cl: 1.30–3.62, *p* < 0.01), and measuring patients’ weight, height, and BMI (OR = 1.75; 95%Cl: 1.06–2.87, *p* < 0.05) were more likely to provide minimal alcohol counseling to patients who consumed alcohol.

### Taking measurements in adult patients

Among the nurses surveyed, 84.6% report that they measure weight on a scale, 75.5% measure height, and 75.8% calculate the BMI of adult patients. Only 64.4% of the nurses measured waist circumference, whereas 76.1% asked about the declared weight of patients (Table 3).

**Table 3:** Measurements of weight, height, BMI in adult patients performed.

Variable	Female
	*n = 331* (%)
Body weight measured on a scale	
Yes	280 (84.6)
No	51 (15.4)
Body weight declared by the patient	
Yes	252 (76.1)
No	79 (23.9)
Body mass index (BMI)	
Yes	251 (75.8)
No	80 (24.2)
Waist circumference	
Yes	213 (64.4)
No	118 (35.6)
Height	
Yes	250 (75.5)
No	81 (24.5)

### Opinions on the nurse’s role as a promoter of healthy lifestyles

Among the study participants, 91.5% believe that nurses are obligated to counsel patients on healthy lifestyles, and 72.5% think they have the knowledge and skills to counsel patients on healthy lifestyles. Also, 88.2% of the nurses believe that healthy lifestyle counseling will be more effective if doctors themselves followed health recommendations. The survey results show that 63.4% of the respondents think that they are effective in helping patients lead healthy lifestyles (Table 4).

**Table 4:** Opinions on the nurse’s role as a promoter of healthy lifestyle.

Opinion	Female
	*n = 331* (%)
A nurse is obliged to provide counseling on a healthy lifestyle among patients
Yes	303 (91.5)
No	28 (8.5)
Patients are more likely to make lifestyle changes after receiving expert counseling from a nurse	
Yes	230 (69.5)
No	101 (30.5)
There are effective strategies and tools designed to help patients with healthy lifestyle	
Yes	186 (56.2)
No	145 (43.8)
I have sufficient knowledge and skills to advise patients on healthy lifestyle
Yes	240 (72.5)
No	91 (27.5)
I’m effective in helping my patients to lead a healthy lifestyle
Yes	210 (63.4)
No	121 (36.6)
Healthy lifestyle counseling will be more effective if a nurse herself follows the health recommendations	
Yes	292 (88.2)
No	39 (11.8)

### Barriers and improvements in the process of assessing, controlling, and guiding the patient

The most common barriers identified by nurses in the process of assessing, monitoring, and guiding patients were the opinion that patients are not interested in improving their diet, physical activity, and weight loss (60.7%), as well as lack of time (51.4%). Another obstacle, according to the respondents, is that patients find it too difficult to change their current habits (54.1%) (Supplementary Table S1). The survey participants think that the three key improvements that would help reduce health problems which are related to diet, physical activity, and weight are simple procedures and guidelines for patient monitoring (59.8%), better tools for communicating information about diet, physical activity, or weight problems to patients or their families (46.5%), as well as more training in assessment and management of a patient’s diet, physical activity and weight control (42.6%) (Supplementary Table S2).

## Discussion

This study is one of the first post-pandemic COVID-19 surveys of nurses working in the primary care sector. It fills information gaps in the literature as regard healthy lifestyle counseling provided by primary care nurses. Additionally, the study assesses various correlates that may influence attitudes toward healthy lifestyle counseling offered to patients among this group of medical professionals.

About 88.5% of the nurses in our survey provided diet and physical activity counseling to their patients. These results indicate the inadequacy of current practices. According to good practice, at each patient visit, the nurse should ask questions regarding potential factors in the development of chronic diseases, especially those that are subject to modification. When making the assessment, you should take into account: the patient’s nutritional preferences in terms of quality and quantity, body weight, and height necessary to calculate and interpret BMI, physical activity, alcohol consumption, and smoking (40).

Other studies have shown that counseling offered by a primary care nurse was more effective than screening. Evidence from other studies also supports the effectiveness of lifestyle interventions made by primary care nurses in relation to chronic disease prevention (e.g., blood pressure, weight, cholesterol levels, diet, and physical activity behaviors, readiness to change and quality of life, and patient satisfaction) (21). Research indicates that some 67.2% of patient encounters with nurses in primary care in Australia consist of disease-specific health education (45).

Nutritional counseling can be used by nurses to achieve health goals and improve nutritional status (46). The findings suggest that nurses understand the importance of nutrition counseling in primary care practice, and provide it to some extent, but its continuation is limited by a number of barriers (47). Physical activity counseling in primary care is inadequate, however, the reasons for this are not well understood. Nurses, like doctors, see physical activity counseling as important. Efforts should be made to improve knowledge and opportunities for physical activity counseling (48). Studies show that the most effective interventions are those combining diet and physical activity (49). However, other studies demonstrate that interventions targeting a single behavior were more effective than mixed ones (50, 51).

Healthy lifestyle counseling and primary prevention have been identified as important strategies for reducing chronic disease prevalence and healthcare costs (52). Both the nurse and the doctor in primary care undertake educational activities and provide guidance on healthy lifestyles (53–55).

Nurses are considered leaders of healthcare teams in undertaking interventions focused on chronic disease management (56). Studies show that activities of primary care nurses can help patients control and lose weight, as well as improve physical activity and eating habits (21, 57). A study conducted in Finland found that nurse-monitored lifestyle change guidance over 3 years resulted in at least a 5% reduction in initial body weight in 18% of overweight people with comorbidities, who were able to maintain their weight for 3 years. Additionally, most of them managed to stabilize their weight after the interventions (58).

These findings emphasize that lifestyle counseling should involve a partnership between the patient and the community nurse based on mutual respect, recognition of the patient as the expert on their current living situation, and the need to involve both parties in the process of lifestyle change (59). The health service should provide patient education and increase the sense of efficacy in maintaining healthier lifestyle habits (59).

Seventy-one percent of the primary care nurses in our study provided minimal anti-smoking interventions to smokers, while 56.8% provided minimal anti-smoking interventions to e-cigarette users. Additionally, 67.7% of the study participants confirmed providing minimal anti-smoking interventions to patients who consume alcohol.

Other publications also indicate that despite the effectiveness of smoking cessation counseling, in practice, the participation of nurses in providing smoking cessation advice is far from satisfactory (60). The most effective smoking cessation strategy is advice and care from nurses (61). In other studies, the majority of nurses agree that identifying and giving alcohol-related advice is a natural part of their job (62). Brief interventions in primary care settings are effective in reducing alcohol consumption, however, implementation has proven difficult (63), as in our study.

The correlates of healthy lifestyle counseling identified in our study (such as sufficient knowledge and skills to advise patients on healthy lifestyle, making measurements of body weight, height, BMI, number of patient visits in a regular working week) are crucial for developing appropriate strategies aimed at improving population health. Additional correlations prove that even though the nurse smokes cigarettes (and she should not) and feels overweight (and she should rather have an optimal BMI and a sense of good weight), this most likely does not constitute a barrier to counseling on a healthy lifestyle. Moreover, a family history of coronary heart disease in the nurse’s family also seems to increase the willingness to provide such counseling.

Nurses’ health behaviors and the impact of these behaviors on professional practice have been identified as important determinants of successful behavior change (64). Nurses who promote healthy behaviors and believe in health promotion are more likely to be positive role models and teach their patients about healthy behaviors (65). It has also been shown that nurses’ health behaviors may constitute a barrier to their health promotion practice (66). Evidence suggests that nurses’ and doctors’ personal body weight is associated with their approach to weight management and their weight management practices (67–69). Similarly, nurses’ personal involvement in physical activity seems to be related to the level of physical activity promotion (70). Systematic reviews suggest that alcohol consumption and personal tobacco use are also associated with health promotion practices (71, 72).

Our survey indicates that the nursing workforce is aging. The largest group of nurses in our survey were women aged 55 and older. This confirms the recent trend of aging medical personnel (the high number of those aged 60 or more) (2).

It has become very important to encourage more young people to study medical faculties to ensure generational replacement in this professional group. Moreover, it is crucial to make the best use of the professional potential of existing medical staff, in accordance with the needs of the system, to support the professional development of current personnel, as well as to prevent the exodus of medical workers abroad (2).

The results of our survey show that 91.5% of nurses believe they have a duty to advise patients on healthy lifestyles. As many as 72.5% of them believe to have sufficient knowledge to do so. Our survey, like many previous studies, showed the need to improve the competence of nurses (61, 73–76). Nurses require training and education to facilitate a patient-centered approach to effective counseling (46).

Professional competence of nurses have proved to be important in building trust in patient–nurse relationship (77). Trained nurses are likely to provide the same or better quality of care and achieve higher levels of patient satisfaction compared to physicians (27). This is an even more important aspect when duties are transferred from a primary care physician to a nurse. Lack of training can make nurses apprehensive about taking on the new duties they are expected to perform (74).

Patients in several reviews experienced greater engagement from nurses than from physicians since nurses devoted them more time in consultations, and it was easier to break social and physical barriers that tend to exist in the case of doctors (34, 78).

Nurses are more likely to identify their own professional group as an educator—more than 80% of nurses undertake patient education, and the time it takes to complete it is usually about 15–20 min (79). Approximately 90% of nurses see the need for further professional training to act as educators (80).

Our study also showed that 88.2% of the participants believe that lifestyle tips could be more effective if nurses themselves led healthy lifestyle.

Promoting a healthy lifestyle is important to protect patients against non-communicable diseases and obesity (81).

Higher quality of life improves the level of health behaviors among nursing staff. Good material situation positively affects their quality of life (82). Nurses often struggle to maintain healthy lifestyles. Although nurses are often considered to have the knowledge needed to participate in health-promoting behaviors, this knowledge may not translate into a lasting change in their health behaviors (83). Another study showed significantly better health-related behaviors related to smoking, fruit/vegetable consumption, and physical activity among nurses than in the general population of medical professionals (84).

In our study, 45.6% of nurses indicated that they practiced physical activity in accordance with WHO recommendations. Similar results were obtained in Poland among working teachers, where 46% of surveyed women met WHO recommendations (85). Similar results were obtained in another study in Poland among white-collar workers, where 42% of women declared moderate activity for at least 150 min a week (86). Data in Poland indicate that 43.9% of Poles and 43.5% of Polish women meet the WHO recommendations regarding the health-promoting dose of PA (physical activity) (87). Higher results were shown in another study, where 75% of nurses declared personal physical activity (88).

In our study, 42.6% of nurses consuming an average of 400 g of vegetables and fruits in their daily diet in at least five portions and more. Lower results were obtained in another study in Poland among remote workers (31.1%), declaring the consumption of vegetables in the amount of 3–5 portions (89). In another study among GPs, only 20% consumed vegetables and fruit in the recommended amounts (41). According to WHO data, only every third Pole follows experts’ recommendations and consumes them 400 g of vegetables and fruit per day (90).

In our study, the prevalence of smoking among nurses was 18.1% and was lower than in the general population in Poland (24%) (91). 28.8% of adult Poles (27.1% of women and 30.8% of men) admitted to smoking every day in 2022 (92). Lower results in Poland were obtained in a survey among doctors, where 7.8% of doctors (including 6.3 women doctors) declared current smoking (93).

In our study, 41.4% of nurses consumed alcohol. Higher results in Poland were obtained among remote workers (80.9%) (94) and the general population in Poland (80%) (95).

Nurses reported lower levels of harmful co-occurring behaviors (smoking and alcohol consumption), as well as higher levels of preventive behaviors (physical activity and fruit/vegetable consumption) compared to the general professional population (84). This is confirmed by the results of another study where 25% of nurses were at risk of risky drinking or disorders related to active alcohol use, and 11% were current smokers (88).

In other studies, nurses reported low levels of physical activity and diet low in vegetables/fruit, abused alcohol (40%), and smoked cigarettes (17%) (96, 97). Nurses are eager to help patients change their lifestyle, considering it an important part of patient care, but some barriers are difficult to overcome (24). This can frustrate nurses and patients, as well as reduce nurses’ empathy for patients and reduce their motivation to advise patients (24).

In our survey, the main barriers to ongoing counseling were the opinion that patients are not interested in improving their diet, physical activity, or weight loss (60.7%) and that it is too difficult for them to change their current habits (54.1%). Also, one in two nurses, though having appropriate training to provide counseling (72.5%), indicated lack of time as one of the main obstacles to ongoing counseling. Lack of time as a barrier to providing dietary advice was more frequently reported among nurses who were overweight (98). The lack of counseling skills is also often mentioned (24). In other studies, the main barriers identified by nurses were lack of motivation, patient factors (24, 99, 100). Some other surveys point to such obstacles as lack of communication skills and lack of knowledge (18, 24, 101). Some nurses reported not knowing enough about specific diet, physical activity, and smoking cessation advice to provide appropriate lifestyle advice (24, 98, 102, 103).

One study found that nurses had inadequate knowledge on obesity treatment, which was a hindrance to their primary care practice in preventing and controlling the disease (104). Other barriers appeared to be a lack of ability to broach the subject, lack of persistence, inability to find common ground with the patient, and insufficient use of existing educational materials (101). Nurses reported barriers at the patient level, including: that patients have limited knowledge about a healthy lifestyle and limited insight into their own behavior (24). According to nurses, patients look for excuses not to give up their habits and they lack motivation to modify their lifestyle (24). The solution to overcoming the existing barriers to primary prevention is to rely on nurses as professionals who are competent to provide patients with healthy lifestyle support (52).

Nurses face potential barriers to leading a healthy lifestyle both in and out of the workplace, including shift work, lack of breaks, fast-paced work environment, and the emotional work of nursing (105). It is important that those providing lifestyle counseling are familiar with patients’ barriers (24). Nurses’ solution to overcome barriers is additional training in counseling skills, which can help them motivate patients (24).

The study has strong points. There are not many papers addressing this topic in other countries, and no such survey has been conducted in Poland either. Lifestyle counseling by nurses was evaluated based on questionnaire data reported by the study participants themselves. Weaknesses of the study. The survey was cross-sectional and conducted at a single time point, which made it impossible to observe changes over longer periods of time. All the assessments were carried out among primary care nurses only, and do not include patients’ opinions in this respect. It should also be noted that the patterns observed among the nurses surveyed may reflect the situation in urban areas. However, all nurses are educated the same way, and their approach to risk factor assessment, counseling, and treatment should be independent of where they work.

## Conclusion

The results of our survey indicate that nurses’ participation in healthy lifestyle counseling in adult patients is unsatisfactory. Efforts should be made to ensure that healthy lifestyle advice is provided at every patient visit.

Further training of nursing staff is required to increase their knowledge on healthy lifestyles. It is important to transfer tasks promoting healthy lifestyles, offering advice and counseling, and promoting healthy lifestyles among primary care patients from the doctor to the nurse.

Interventions in primary care should be designed based on the specific obstacles nurses may face in leading healthy lifestyles. Further research using identified facilitators and barriers to promoting healthy lifestyles in primary care patients with chronic diseases can help design future training programs for nurses. Employers should also consider removing barriers that make it difficult for nurses to promote healthy lifestyles.

Future research should also evaluate ways to increase nurses’ willingness to offer lifestyle counseling, assess specific resources and tools to help with counseling and identify best practices for communication when providing the aforementioned counseling.

## Data availability statement

The original contributions presented in the study are included in the article/Supplementary material, further inquiries can be directed to the corresponding author.

## Ethics statement

The studies involving humans were approved by the Bioethics Committee of the Medical University of Lodz (RNN/315/18/KE). The studies were conducted in accordance with the local legislation and institutional requirements. The participants provided their written informed consent to participate in this study.

## Author contributions

MZ: Conceptualization, Data curation, Formal analysis, Investigation, Methodology, Software, Supervision, Validation, Writing – original draft, Writing – review & editing. SK: Supervision, Writing – review & editing. DK: Supervision, Writing – review & editing.
