# Supplementary material for: Nurse-led lifestyle counseling in Polish primary care: the effect of current health status and perceived barriers
Source: Front Public Health. 2024 Feb 19;12:1301982. doi: 10.3389/fpubh.2024.1301982 (PMC10910074; doi:10.3389/fpubh.2024.1301982)
Supplement: Supplementary file 1 [file Table_1.DOCX]

Questionnaire number:

The questionnaire is anonymous and the results of the research will be used for scientific purposes only. I would like to ask you to answer all the following questions. A single answer should be given – unless the information attached to the question indicates another possibility.

| Section A. Characteristics of the treated patient population |
| --- |

**A1. Please specify your patient group.**

*Please select one answer per line.*

|  | Yes | No |
| --- | --- | --- |
| 1. Children up to 2 years of age |  |  |
| 1. Children aged 2-11 years |  |  |
| 1. Youth aged 12-17 |  |  |
| 1. Adults aged 18-29 |  |  |
| 1. Adults aged 30-49 |  |  |
| 1. Adults aged 50-65 |  |  |
| 1. People over 65 years of age |  |  |

**A2. During a routine examination of a patient:**

*Please select one answer per line.*

|  | Never | Seldom | Sometimes | Often | Always |
| --- | --- | --- | --- | --- | --- |
| 1. How often do you assess the patient's diet or physical activity? |  |  |  |  |  |
| 1. How often do you ask  about the patient's smoking or alcohol consumption |  |  |  |  |  |
| 1. According to the current health policy, taking into account the adult population - how often do you recommend: | | | | | |
| - Healthy Diet/Proper Nutrition |  |  |  |  |  |
| - Physical activity |  |  |  |  |  |

**A3. For patients who do NOT have chronic conditions caused by being overweight, and who eat an unhealthy diet, are not physically active or obese:**

How often do you….

|  | NEVER | SELDOM | SOMETIMES | OFTEN | ALWAYS |
| --- | --- | --- | --- | --- | --- |
| 1. Gives general advice on changing your diet, physical activity, or losing weight? |  |  |  |  |  |
| 1. It provides detailed advice on: | | | | | |
| Diet/nutrition (e.g. "Eat more fruits and vegetables" or "Increase your calcium diet")? |  |  |  |  |  |
| Physical activity (e.g. "Please reduce the amount of time you sit e.g. in front of the TV, in front of the computer", "Please increase your activity by taking daily walks")? |  |  |  |  |  |
| Weight management (e.g., lose X amount of weight by reducing the calorie content of your meals or by exercising)? |  |  |  |  |  |
| Refer these patients to another health professional or offer to participate in programs outside of your practice for further evaluation and/or management? |  |  |  |  |  |
| Systematically observes/tracks patient behavior or other measures of progress regarding diet, physical activity and body weight ? |  |  |  |  |  |

**A4. For patients who HAVE chronic conditions caused by being overweight and who eat an unhealthy diet, are not physically active or obese:**

**How often do you.....**

|  | NEVER | SELDOM | SOMETIMES | OFTEN | ALWAYS |
| --- | --- | --- | --- | --- | --- |
| 1. Gives general advice on changing your diet, physical activity, or losing weight? |  |  |  |  |  |
| 1. It provides detailed advice on: | | | | | |
| Diet/nutrition (e.g. "Eat more fruits and vegetables" or "Increase your calcium diet")? |  |  |  |  |  |
| Physical activity (e.g. "Please reduce the amount of time you sit e.g. in front of the TV, in front of the computer", "Please increase your activity by taking daily walks") ? |  |  |  |  |  |
| Weight management (e.g., lose X amount of weight by reducing the calorie content of your meals or by exercising)? |  |  |  |  |  |
| Refer these patients to another health professional or offer to participate in programs outside of your practice for further evaluation and/or management? |  |  |  |  |  |
| Systematically observes/tracks patient behavior or other measures of progress regarding diet, physical activity and body weight ? |  |  |  |  |  |

**A5. How often do you evaluate the following variables in your patients?**

*Please tick all the points that apply*

|  | With each routine visit | With each visit | Once a year | If clinically indicated | Never | A different answer  (please specify) |
| --- | --- | --- | --- | --- | --- | --- |
| 1. Body weight measured on the scale |  |  |  |  |  |  |
| 1. Patient's declared body weight |  |  |  |  |  |  |
| 1. Body Mass Index (BMI) |  |  |  |  |  |  |
| 1. Waist circumference |  |  |  |  |  |  |
| 1. Height |  |  |  |  |  |  |

**The next questions concern nursing practice related to children and adolescents (aged 2-17 years).**

**A6. How often do you evaluate the following variables in adolescent patients or children (aged 2-17 years)?**

*Please tick all the points that apply*

|  | With each routine visit | With each visit | Once a year | If clinically indicated | Never | Other  (what?) |
| --- | --- | --- | --- | --- | --- | --- |
| 1. Body weight measured in the doctor's office |  |  |  |  |  |  |
| 1. Body height measured in the doctor's office |  |  |  |  |  |  |
| 1. Body Mass Index (BMI) |  |  |  |  |  |  |
| 1. Waist circumference or ratio of waist circumference to hip circumference |  |  |  |  |  |  |
| 1. Weight-to-age ratio using a percentile chart |  |  |  |  |  |  |
| 1. Height-to-Age Ratio Using Percentile Chart |  |  |  |  |  |  |
| 1. BMI in relation to age using the percentile chart |  |  |  |  |  |  |

**A7. In your daily nursing practice, do you treat your patients:**

*Please tick all the points that apply*

|  | Yes, every time | Yes, in more than half of the cases | Yes, in less than half of the cases | Never |
| --- | --- | --- | --- | --- |
| 1. Do you ask the patient about smoking during the interview? |  |  |  |  |
| 1. Do you record in your medical records information about the patient's smoking? |  |  |  |  |
| 1. When taking an interview, do you ask the patient about the use of e-cigarettes? |  |  |  |  |
| 1. Do you record information in your medical records about the use of e-cigarettes by the patient? |  |  |  |  |
| 1. Do you ask the patient about their alcohol consumption while taking a medical history? |  |  |  |  |
| 1. Do you record information about alcohol consumption in your medical records? |  |  |  |  |
| 1. Do you provide minimal smoking intervention to patients who smoke? |  |  |  |  |
| 1. Do you provide minimal smoke-free intervention to patients who use e-cigarettes? |  |  |  |  |
| 1. Do you provide minimal alcohol intervention to patients who consume alcohol? |  |  |  |  |
| 1. Do you suggest a referral to a smoking clinic for a patient who smokes cigarettes? |  |  |  |  |
| 1. Do you suggest a referral to an anti-smoking clinic for a patient who uses e-cigarettes? |  |  |  |  |
| 1. Do you suggest a referral to an addiction clinic for a patient who abuses alcohol? |  |  |  |  |

| Section B. Barriers to Patient Assessment, Control, and Management |
| --- |

**B1. Of the following factors, please identify the 3 main barriers to patient assessment and/or management in relation to diet, physical activity and weight management.**

*Please highlight the 3 main barriers*

| 1. Lack of time |  |
| --- | --- |
| 1. It is not my responsibility |  |
| 1. I'm not properly trained in this area |  |
| 1. It's too difficult to evaluate and manage |  |
| 1. Inadequate reimbursement |  |
| 1. Lack of appropriate referrals for consultation in terms of diet, physical activity and body weight |  |
| 1. Patients are not interested in improving their diet, physical activity, and weight loss |  |
| 1. Fear of offending the patient |  |
| 1. It is too difficult for patients to change their habits |  |
| 1. Lack of effective tools and information to provide to patients |  |
| 1. Lack of effective treatments |  |

**B2. In the context of your nursing practice, what are THREE key improvements that would help reduce diet, physical activity, and weight-related health issues?**

*Please highlight the top 3 improvements*

| 1. Ways to Better Identify Problems Related to Poor Diet, Low Physical Activity and Overweight in Patients |  |
| --- | --- |
| 1. Simple Procedures and Guidelines for Patient Monitoring |  |
| 1. Higher reimbursement for this type of counseling |  |
| 1. Better tools to communicate diet, physical activity or weight problems to patients or their families |  |
| 1. Better mechanisms for referring patients to specific medical advice |  |
| 1. More training for healthcare professionals on the assessment and management of diet, physical activity, and weight management |  |
| 1. More training for you on the assessment and management of diet, physical activity and weight control |  |
| 1. Better information systems for archiving medical records and tracking treatment progress |  |
| 1. Better information systems to identify relevant services |  |
| 1. Better mechanisms to connect patients to specific medical services |  |

**B3. Please specify to what extent you agree with the following statements:**

*Please select one answer per line.*

|  | I strongly agree | I tend to agree | I don't have an opinion | I tend to disagree | I strongly disagree |
| --- | --- | --- | --- | --- | --- |
| 1. Nurses have a duty to promote among their patients: | | | | | |
| - healthy eating |  |  |  |  |  |
| - adequate physical activity |  |  |  |  |  |
| - maintaining a healthy weight or losing weight |  |  |  |  |  |
| 1. Patients are more likely to make lifestyle changes after receiving expert advice from a nurse |  |  |  |  |  |
| 1. There are effective strategies and tools in place to help patients with: | | | | | |
| - health eating |  |  |  |  |  |
| - adequate physical activity |  |  |  |  |  |
| - maintaining a healthy weight or losing weight |  |  |  |  |  |
| 1. I am convinced that I have the right skills to advise my patients on: | | | | | |
| - health eating |  |  |  |  |  |
| - adequate physical activity |  |  |  |  |  |
| - maintaining a healthy weight or losing weight |  |  |  |  |  |
| 1. I effectively help my patients with: | | | | | |
| - health eating |  |  |  |  |  |
| - adequate physical activity |  |  |  |  |  |
| - maintaining a healthy weight or losing weight |  |  |  |  |  |
| 1. In order to effectively encourage patients to lead a healthy lifestyle, the nurse must lead such a lifestyle herself | | | | | |
| A nurse will provide more credible and effective counseling if she or she will: | | | | | |
| - eat healthily |  |  |  |  |  |
| - maintain adequate physical activity |  |  |  |  |  |
| - maintain a healthy body weight or lose weight |  |  |  |  |  |

| Section C. Health Status/Health Behaviors |
| --- |

**C1. How do you assess your overall health?**

*Please select one answer*

| Like: | Perfect | Very good | Good | Moderately good | Disappointing |
| --- | --- | --- | --- | --- | --- |
|  |  |  |  |  |  |

**C2. Do you suffer from chronic diseases related to overweight or obesity (e.g. cardiovascular diseases, type II diabetes, cardiovascular diseases, etc.)?**

Yes

No

**C3. Are you overweight or obese?**

Yes

No

**C4. Are you being treated for any of the following conditions?**

*Please tick all the points that apply*

|  | No | Yes |
| --- | --- | --- |
| 1. Abnormal body mass index (BMI) |  |  |
| 1. Abnormal lipid profile |  |  |
| 1. Hypertension |  |  |
| 1. Eating disorders such as anorexia or bulimia |  |  |
| 1. Asthma |  |  |
| 1. Type II diabetes |  |  |
| 1. Coronary artery disease |  |  |
| 1. Neoplastic disease |  |  |
| 1. Arthritis |  |  |
| 1. Apnea |  |  |
| 1. Chronic obstructive pulmonary disease |  |  |
| 1. Pain |  |  |

**C5. Have you had any of the following diseases in your family?**

|  | No | Yes |
| --- | --- | --- |
| 1. Diabetes |  |  |
| 1. Coronary artery disease |  |  |
| 1. Neoplastic disease |  |  |

**C6. The following questions are about the nutrients you are consuming in the last 30 days.**

**How many meals a day do you usually eat?**

(fruit, cake, bread, etc. are meals, drinks and candy are not meals)

1 2 3 4 5 6 more than 6

**What type of bread do you choose most often?**

*Please select one or more answers*.

| 1. Light or dark rye bread |  |
| --- | --- |
| 1. Whole grain rye bread |  |
| 1. White bread |  |
| 1. White bread, coarse grain |  |
| 1. White bread, Italian |  |

**What type of fat do you use to spread on your bread?**

*Please select one or more answers.*

| 1. Nothing/ No |  |
| --- | --- |
| 1. Reduced-fat margarine |  |
| 1. Plant-based margarine |  |
| 1. Butter |  |
| 1. Butter and margarine mixture |  |
| 1. Lard |  |

**How often have you eaten the following products with bread in the last week?**

| **0** | **1-2** | **3-4** | **5-7** | **frequency/week** |
| --- | --- | --- | --- | --- |
| **􀂅** | **􀂅** | **􀂅** | **􀂅** | Cheese 0%-17% fat |
| **􀂅** | **􀂅** | **􀂅** | **􀂅** | Cheese 27%-38% fat |
| **􀂅** | **􀂅** | **􀂅** | **􀂅** | Meat |
| **􀂅** | **􀂅** | **􀂅** | **􀂅** | Fish at least 200g |
| **􀂅** | **􀂅** | **􀂅** | **􀂅** | Eggs |
| **􀂅** | **􀂅** | **􀂅** | **􀂅** | Salad with mayonnaise |
| **􀂅** | **􀂅** | **􀂅** | **􀂅** | Vegetables |
| **􀂅** | **􀂅** | **􀂅** | **􀂅** | Marmalade/honey |

**How often did you eat the following types of hot meals in the past week?**

| **0** | **1-2** | **3-4** | **5-7** | **Frequency/week** |
| --- | --- | --- | --- | --- |
| **􀂅** | **􀂅** | **􀂅** | **􀂅** | Beef/veal |
| **􀂅** | **􀂅** | **􀂅** | **􀂅** | Pork |
| **􀂅** | **􀂅** | **􀂅** | **􀂅** | Poultry |
| **􀂅** | **􀂅** | **􀂅** | **􀂅** | Fishes |
| **􀂅** | **􀂅** | **􀂅** | **􀂅** | Offal/ giblets |
| **􀂅** | **􀂅** | **􀂅** | **􀂅** | Egg dishes |
| **􀂅** | **􀂅** | **􀂅** | **􀂅** | Vegetables/vegetarian dishes |
| **􀂅** | **􀂅** | **􀂅** | **􀂅** | Porridge |
| **􀂅** | **􀂅** | **􀂅** | **􀂅** | Ready meals |
| **􀂅** | **􀂅** | **􀂅** | **􀂅** | Pizza/hamburgers |
| **􀂅** | **􀂅** | **􀂅** | **􀂅** | Sausages, etc. |

**What kind of fat do you use for cooking/frying**?

*Please select one or more answers.*

| 1. Nothing/ No |  |
| --- | --- |
| 1. Margarine |  |
| 1. Vegetable margarine |  |
| 1. Butter |  |
| 1. A mixture of vegetable fats |  |
| 1. Lard |  |
| 1. Canola oil |  |
| 1. Olive oil |  |
| 1. Corn oil/ sunflower oil/ grape seed oil |  |
| 1. Other |  |

**How often have you eaten potatoes/pasta/rice, etc., in the last week? as an addition to hot dishes?**

| **0** | **1-2** | **3-4** | **5-7** | **Frequency/week** |
| --- | --- | --- | --- | --- |
| **􀂅** | **􀂅** | **􀂅** | **􀂅** | Potatoes |
| **􀂅** | **􀂅** | **􀂅** | **􀂅** | Pasta |
| **􀂅** | **􀂅** | **􀂅** | **􀂅** | Rice/groats/ bulgur groats, etc. |
| **􀂅** | **􀂅** | **􀂅** | **􀂅** | Bread |
| **􀂅** | **􀂅** | **􀂅** | **􀂅** | Other |

**How often have you eaten raw or cooked vegetables as a side dish in the last week?**

| **0** | **1-2** | **3-4** | **5-7** | **Frequency/week** |
| --- | --- | --- | --- | --- |
| **􀂅** | **􀂅** | **􀂅** | **􀂅** | Salad or raw vegetables |
| **􀂅** | **􀂅** | **􀂅** | **􀂅** | Ready-made vegetables |
| **􀂅** | **􀂅** | **􀂅** | **􀂅** | Hot vegetables |
| **􀂅** | **􀂅** | **􀂅** | **􀂅** | Other |

**How much fruit do you usually eat per day and week?**

*1 serving = 1 piece of fruit, e.g. an apple or 1 piece, e.g. watermelon*

| 1. I don't eat fruit |  |
| --- | --- |
| 1. 1-2/week |  |
| 1. 3-4/week |  |
| 1. 5-6/week |  |
| 1. 1-2/week |  |
| 1. 3-4/week |  |
| 1. 5-6/week |  |
| 1. More than 6 times/day |  |

**C7. How many litres (on average) do you drink of pure mineral/spring water per day?**

*Please select one answer*

About 2 cups (up to half a pint)

From 0.5 to 1liter

1.5 liters (large bottle)

2 liters and more

I don't drink

**C8. Physical activity. The following questions apply to physical activity as part of work**

**a. Please indicate the number of days in the last week (7 days) in which you have performed intense physical exertion, e.g. lifting heavy objects, digging, construction work, climbing stairs – as part of your professional job.**

________ days a week

I haven't done that→ →→ → go to question C8b

**Please indicate how much time you usually devote to intense physical activity as part of your work on one of these days.**

________ hours ________minutes PER DAY

**Please indicate how many floors you climb per day**

**________**number of floors

**b. Please indicate the number of days in the last week (7 days) in which you have engaged in moderate physical activity, e.g. carrying light items, cycling at a normal pace, as part of your job. Please do not consider walking.**

**________** days a week

**Please indicate how much time you usually devote to moderate physical activity as part of your professional activity on one of these days.**

________ hours ________minutes PER DAY

**c. Please indicate the number of days in the last week (7 days) in which you have walked for at least 10 minutes during your professional life. Please do not include the time it takes to get to and from work.**

________ days a week

I haven’t done that. → →→ → go to question C9

**Please indicate how much time you spend walking on one of these days as part of your professional career.**

_____ hours ______ minutes PER DAY

**C9. Physical activity in your free time. The following questions apply to physical activity outside of work**

**a. Have you performed activities that require intense physical exertion (lifting heavy objects, digging the ground, aerobics, fast running, fast cycling, fast swimming) in your free time in the last 7 days that lasted at least 10 minutes straight?**

Yes – for how many days in the last week? ................... days

No please go to question C9b

I don't know/I'm not sure, please go to question C9b

**On average, how much time you performed activities that required intense physical effort during such a day that lasted at least 10 minutes continuously?**

............. minutes per day

I don't know/I'm not sure

**b. Have you done activities that require moderate to moderate physical exertion (e.g. carrying lighter weights, cycling at a normal pace, playing volleyball or very fast walking) that have lasted at least 10 minutes without a break in the last 7 days?**

Yes – for how many days in the last week? ................... days

No, please go to question C9c

I don't know/I'm not sure, please go to question C9c

**On average, how much time did you perform activities that require moderate physical effort during such a day?**

............. minutes per day

I don't know/I'm not sure

**c. Have you walked (walking down the street, e.g. shopping, to work, walking) for at least 10 minutes in the last 7 days? Continuously?**

Yes – for how many days in the last week? ................... days

Do not go to question C9d

I don't know/I'm not sure, go to question C9d

**On average, how much time did you spend walking or walking during such a day?**

............. minutes per day

I don't know/I'm not sure

**d. How do you usually get from home to work and back?**

I walk or ride a bike;

I commute to work and return by car, please go to question C10

by public transport please go to question C10

I don't work or I work from home please go to question C10

**e. How many minutes a day does it take you to walk or bike to work and back home?**

less than 15 minutes

15 to 30 minutes

more than 30 to 60 minutes

more than an hour

**C10. The questions relate to the time you spend sitting at work, at home and in your leisure time. This can include time spent sitting at a desk, reading, watching TV, etc.**

a. Please state how much time you spent sitting PER DAY on weekdays of the last week (7 days).

......... hours..........minutes PER DAY

including in the course of professional work

......... hours..........minutes PER DAY

b. Please indicate how much time you have spent sitting PER DAY on your public holidays in the last week (7 days).

......... hours..........minutes PER DAY

c. On average, how many hours a day do you spend watching TV shows?

......... hours..........minutes PER DAY

d. How many hours a day do you spend on average working with a computer (including games, internet, etc.) outside of work?

......... hours..........minutes PER DAY

e. How many hours a day do you spend working with a computer on average in your professional work?

......... hours..........minutes PER DAY

**C11. How tall are you (without shoes)?**

Cm

**C12. What is your current body weight (without shoes)?**

Kg

**C12a. If you are currently pregnant, how much was your body weight before pregnancy?**

Kg

**C13. Are you currently trying to:**

Lose weight

Gain weight

Maintain your current weight

I'm not trying to change anything

**C14. Year of Birth**

1

9

year

**C15. Sex**

*Please select one answer*

Woman Man

**C16. Do you smoke?**

*Please select one answer*

Yes No ………………….. go to question C22

**C17. How many cigarettes do you smoke a day?**

Number of cigarettes smoked per day

**C18. What type of cigarettes do you currently smoke most often?**

with filter

without filter

hand-twisted

slim

menthol

others, what?............

**C19. Would you like to quit smoking?**

*Please select one answer*

I'm going to quit smoking in the next month

I'm considering quitting in the next 12 months

I will quit smoking, but not in the next 12 months.

I'm not going to quit smoking

*I do not know*

**C20. Have you ever tried to quit smoking?**

no go to question C22

yes (how many times................)

**C21. When was the last time you tried to quit smoking, i.e. you didn't smoke for at least 24 hours?**

in the last month

over 1 month to half a year ago

over half a year to 1 year ago

over 1 year ago

**C22. Are you exposed to so-called secondhand smoke, i.e. inhaling cigarette smoke at home or at work?**

Yes, only at home

Yes, only at work

Yes, at home and at work

Yes, in other situations

No

**C23. How many hours a day do you spend in rooms where someone smokes?**

I don't stay in such rooms at all

less than 1 hour per day

from 1 hour to 5 hours per day

5 to 8 hours a day

more than 8 hours a day

**C24. Have you ever used e-cigarettes (even once)?**

yes

no please go to question C28

**C25. Do you currently use e-cigarettes?**

Yes, every day

Yes, occasionally

I don't use at all please go to question C28

**C26. Do you use nicotine-containing e-cigarettes?**

Yes

No

I do not know

**C27. How often do you use an e-cigarette per day?**

once a day

2-5 times a day

6-10 times a day

11-20 times a day

more than 20 times a day

**C28. How many drinks have you had in the last 7 days?***(1 drink = 1 glass of wine, 1 small shot of vodka, 1 beer):*

I drank /am .......... number of vodka drinks (cognac, brandy, etc.)

I drank /am .......... number of wine drinks

I drank /am .......... number of beer drinks

Not applicable

**C29. Which of the following best describes your primary care nursing practice? Are you...**

*Please tick all the points that apply*

| 1. Owner or co-owner of a private nursing practice |  |
| --- | --- |
| 1. An employee of a private nursing practice |  |
| 1. An employee in a large medical group or in a private health care system |  |
| 1. An employee employed in an independent public health care institution (SPZOZ) |  |

**C30. Please indicate the number of patient visits during the routine work week (except for out-of-turn patients in unplanned emergencies).**

Number of patient visits

I do not know

| Section D. Personal Qualities |
| --- |

**D1. How long have you been working at this plant?**

................... Years or months (please select one)
