# Supplementary material for: Nurse-led lifestyle counseling in Polish primary care: the effect of current health status and perceived barriers
Source: Front Public Health. 2024 Feb 19;12:1301982. doi: 10.3389/fpubh.2024.1301982 (PMC10910074; doi:10.3389/fpubh.2024.1301982)
Supplement: Supplementary file 2 [file Table_2.DOCX]

**Table S1.** Barriers in the process of assessing, controlling, and guiding the patient

| **Barriers** | **Total**  *n=331* (%) |
| --- | --- |
| Lack of time | 170 (51.4) |
| Patients are not interested in improving their diet, physical activity, and weight loss | 201 (60.7) |
| Patients find it too difficult to change their current habits | 179 (54.1) |

**Table S2.** Improvements that would help reduce health problems dependent on diet, physical activity, and body weight

| **Improvements** | **Total**  *n=331* (%) |
| --- | --- |
| Simple procedures and guidelines for patient monitoring | 198 (59.8) |
| Better tools to communicate information about diet, physical activity, or weight problems to patients or their families | 154 (46.5) |
| More training for healthcare professionals in the assessment and management of diet, physical activity, and patient weight management | 141 (42.6) |
